# Maternal Large Yellow Tea Supplementation Confers Intergenerational Protection Against BPA-Induced Metabolic and Behavioral Disorders in Mice

**DOI:** 10.3390/metabo16080588

**Published:** 2026-08-18

**Authors:** Erkang Jiang, Hongyu Wang, Meiyun Li, Xi Wang, Guohuo Wu, Shoujun Huang, Huijun Cheng, Zhuang Li, Zhongwen Xie

**Affiliations:** 1 School of Life Science, Anhui Agricultural University, 130 Changjiang West Road, Hefei 230036, China; 20721550@stu.ahau.edu.cn (H.W.); 25723272@stu.ahau.edu.cn (M.L.); 25723259@stu.ahau.edu.cn (X.W.); huangshoujun@ahau.edu.cn (S.H.); 2 National Key Laboratory of Tea Plant Germplasm Innovation and Resource Utilization, School of Tea Sciences, 130 Changjiang West Road, Hefei 230036, China; wuguohuo@fynu.edu.cn (G.W.); 23113@ylnu.edu.cn (H.C.); 3 Center for Biotechnology, Anhui Agricultural University, 130 Changjiang West Road, Hefei 230036, China; zhuangli100313@ahau.edu.cn

**Keywords:** large yellow tea, bisphenol A, cross-generational protection, bidirectional metabolic regulation, hyperactivity, excitation-inhibition balance

## Abstract

**Background:** Large yellow tea (LYT), a distinctive variety made from mature leaves, has recently gained attention for its remarkable health benefits. However, whether these benefits can be transmitted from mother to offspring remains unexplored. **Purpose:** This study investigated whether maternal LYT consumption confers intergenerational protection against metabolic and behavioral disorders induced by perinatal bisphenol A (BPA) exposure in F1 offspring. **Methods:** A mouse model of perinatal BPA exposure (0.03% in diet) was established with or without maternal LYT supplementation (2.5% in diet). Metabolic parameters were assessed through biochemical assays and gene expression analysis (RT-PCR). Energy expenditure and spontaneous activity were monitored using a Comprehensive Lab Animal Monitoring System (CLAMS). Hippocampal proteomic profiling was performed using label-free quantitative proteomics. **Results:** LYT significantly reduced maternal BPA body burden, potentially via limiting absorption, enhancing glucuronidation metabolism, and promoting excretion. Notably, LYT exhibited bidirectional metabolic regulation, alleviating gestational hyperglycemia in dams while restoring hypoglycemia in offspring, and normalizing underweight and hypolipidemia. Mechanistically, the SIRT6 (sirtuin 6)/FOXO1 and SIRT6/SREBP1 pathways may be involved in regulating gluconeogenesis and lipogenesis. Concurrently, LYT rectified BPA-induced hyperactivity and reduced excessive energy expenditure. Proteomic analysis revealed that LYT partially restores BPA-induced dysregulation of cholesterol metabolism and glutamatergic/GABAergic synaptic pathways, which may contribute to rebalancing synaptic homeostasis. **Conclusions:** These findings suggest that maternal LYT supplementation confers intergenerational protection against BPA-induced metabolic and behavioral disorders in mice, potentially acting through enhanced toxin clearance, bidirectional metabolic regulation, behavioral normalization, and partial restoration of hippocampal synaptic homeostasis. This study provides a theoretical basis for developing natural dietary interventions to mitigate developmental toxicant-induced health risks.

## 1. Introduction

Large yellow tea (LYT) is a traditional Chinese tea made from mature leaves (“one bud with three to six leaves”) [1,2]. Its unique yellowing process modifies the chemical profile, contributing to bioactivities distinct from those of green teas. Previous studies from our laboratory have demonstrated that LYT exerts significant anti-hyperglycemic effects in high-fat-diet-fed mice [3] and ameliorates metabolic syndrome in db/db mice [4]. However, these investigations were confined to adult animals, leaving a critical gap: whether the health benefits of LYT can be transmitted from mother to offspring.

This question is particularly relevant to the Developmental Origins of Health and Disease (DOHaD) paradigm, which posits that early-life exposures profoundly influence long-term health outcomes [5,6]. The perinatal period represents a window of heightened susceptibility, with toxicant exposure linked to metabolic and neurodevelopmental disorders later in life [7,8].

Bisphenol A (BPA), a ubiquitous endocrine-disrupting chemical used in polycarbonate plastics and epoxy resins, exemplifies such environmental threats [9]. Perinatal BPA exposure is implicated in metabolic disorders—including obesity and diabetes [10,11,12,13]—as well as hyperactivity [14], with divergent phenotypes (hyper/hypoglycemia, obesity/leanness) reported depending on exposure conditions [15]. Human exposure to BPA is virtually unavoidable through contaminated food, water, and air, leading to widespread public health concerns regarding its toxic effects.

Given the widespread exposure to BPA and its documented health impacts, identifying dietary interventions that could mitigate its toxicity represents a significant public health priority. Tea and its constituents, particularly epigallocatechin gallate (EGCG), have shown promise in counteracting BPA-induced toxicity in cellular and animal models [15,16,17]. However, whether whole LYT—with its complex mixture of catechins, theanine, caffeine, and polysaccharides—could confer intergenerational protection against BPA has not been investigated.

Here, we established a mouse model of perinatal BPA exposure and tested whether maternal dietary LYT supplementation confers intergenerational protection against BPA-induced metabolic and behavioral disorders. In this study, “intergenerational protection” refers to the protective effect of maternal intervention on directly exposed F1 offspring. True transgenerational effects (requiring F2 and subsequent generations without direct exposure) are beyond the scope of this work.

Our findings reveal that LYT acts as a natural BPA antagonist through multiple potential mechanisms: enhancing toxin clearance, bidirectionally regulating glucose and lipid metabolism, correcting hyperactivity, and partially restoring synaptic homeostasis in the hippocampus.

## 2. Materials and Methods

### 2.1. Preparation and Chemical Analysis of Large Yellow Tea Extract

LYT samples were provided by Bao Er Zhong Xiu Tea Industry Co., Ltd. (Lu’an, China). Water-soluble components were prepared as previously described [4]. Briefly, tea powder (50 g) was boiled in ultra-pure water under stirring at 85 °C for 30 min, followed by ultrasonic extraction at 75 °C for 30 min (100 W average power). The extract was filtered, concentrated, and lyophilized.

Catechin and caffeine contents were analyzed using a Waters Ultra Performance Liquid Chromatography (UPLC) system equipped with a 600 controller and 2489 UV/Visible detector, following our established protocol [18]. Amino acid composition was determined using a HITACHI L-8900 automatic amino acid analyzer. Polysaccharide content was measured by the phenol-sulfuric acid method.

### 2.2. Animals and Experimental Design

CD-1 mice (female, *n* = 18; male, *n* = 9) were purchased from the National Resource Center of Model Mice (NRCMM, Nanjing, China). Mice were housed under specific pathogen-free (SPF) conditions at the Laboratory Animal Center of Anhui Agricultural University, with constant temperature (22 ± 1 °C), humidity (50 ± 5%), and a 12:12 h light-dark cycle (lights on at 8:00 a.m.). All animals had free access to water and food.

After one week of acclimatization, female mice were randomly assigned to three dietary groups (*n* = 6 litters per group): Control group: modified AIN93 diet with 7% corn oil replacing soybean oil; BPA group: modified AIN93 diet supplemented with 0.03% BPA (≥99.0% purity, MACKLIN, Shanghai, China); BPA + LYT group: modified AIN93 diet supplemented with 0.03% BPA and 2.5% LYT. Male mice were maintained on the control diet. All diets were prepared by Trophic Animal Feed High-Tech Co., Ltd. (Nantong, China). After five weeks of dietary intervention, females were mated with males (female:male = 2:1). Females continued on their respective diets throughout gestation and lactation. After weaning, offspring were maintained on the control diet. One female and one male pup from each litter were randomly selected for subsequent experiments, with litter treated as the independent experimental unit to avoid litter effect bias. The 2.5% LYT dosage in the diet was selected based on body surface area normalization, which is approximately equivalent to 10 g of daily tea consumption in human adults, a level achievable through regular dietary intake. The 0.03% BPA dosage was chosen based on previous studies to induce clear metabolic and behavioral phenotypes in offspring, corresponding to high-level environmental exposure scenarios. Food and water intake were monitored daily. Maternal food and energy intake were recorded throughout gestation and lactation; no significant differences were observed among the three groups, excluding maternal nutritional status as a confounding factor for offspring phenotypes. Body weight and fasting blood glucose (measured from tail vein using Nova StatStrip Xpress Glucose Meter) were recorded weekly. At 24 weeks of age, offspring mice were fasted for 12 h and euthanized by spinal dislocation. Liver tissues were collected for gene expression analysis, with portions preserved in RNAlater or flash-frozen in liquid nitrogen for subsequent assays.

### 2.3. BPA Quantification

Urine and feces were collected from maternal mice housed in metabolic cages for 48 h. Free BPA in serum, urine, and feces was extracted with acetonitrile and analyzed using a Waters ACQUITY UPLC H-Class System with fluorescence detection (excitation: 276 nm, emission: 333 nm). Separation was performed on a CORTECS UPLC C18+ column (2.1 × 100 mm, 1.6 μm) at 30 °C with acetonitrile:water (50:50, *v*/*v*) mobile phase at 0.4 mL/min. Total BPA (free plus conjugated) was measured following enzymatic hydrolysis with β-glucuronidase (Shanghai Yuanye Bio-Technology Co., Ltd., Shanghai, China) in ammonium acetate buffer (pH 5) overnight at 37 °C. Conjugated BPA was calculated as total BPA minus free BPA.

### 2.4. Serum Biochemical Parameters

Serum was collected by centrifugation at 3000 rpm for 5 min at 4 °C. Triglyceride (TG), total cholesterol (TC), and low-density lipoprotein cholesterol (LDL-C) were measured using commercial kits (Nanjing Jiancheng Bioengineering Institute, Nanjing, China). Glucagon, insulin, glucagon-like peptide-1 (GLP-1), and leptin were quantified by enzyme-linked immunosorbent assay (ELISA) kits (Shanghai Jianglai Biotech, Shanghai, China) according to the manufacturer’s instructions. Hepatic glycogen content was measured as previously reported with minor modifications [19].

### 2.5. Metabolic and Behavioral Analyses

At 20 weeks of age, offspring mice were evaluated using a Comprehensive Lab Animal Monitoring System (CLAMS; Columbus Instruments, Columbus, OH, USA). Oxygen consumption (VO_2_), carbon dioxide production (VCO_2_), energy expenditure, spontaneous activity (ambulatory, total horizontal, and vertical movements), and food intake were monitored continuously for 72 h. Data were collected at 20-min intervals. Respiratory exchange ratio (RER = VCO_2_/VO_2_) was calculated for metabolic substrate analysis. Body composition (fat mass, lean mass, and fluid mass) was assessed by nuclear magnetic resonance (NMR; Minispec LF90II; Bruker Optics, Billerica, MA, USA) in conscious mice following CLAMS measurements.

### 2.6. Quantitative Real-Time PCR Analysis

Total RNA was extracted from liver tissues using RNA isolator (Vazyme Biotech, Nanjing, China). Reverse transcription was performed with the HiScript^®^ II 1st Strand cDNA Synthesis Kit (Vazyme). Real-time PCR was conducted using AceQ qPCR SYBR Green Master Mix (Vazyme) on a Bio-Rad CFX System. Primer sequences are provided in Supplementary Table S1. Relative gene expression was calculated using the 2^−ΔΔCt^ method with β-actin as the internal control.

### 2.7. Label-Free Quantitative Proteomic Analysis of Hippocampus

Hippocampal tissues from female offspring mice (*n* = 3 biological replicates per group, each from an independent litter) were subjected to label-free quantitative proteomics. Protein extraction was performed using SDT lysis buffer, followed by protein quantification with BCA assay. Protein samples were digested with trypsin using the filter-aided sample preparation (FASP) method, and peptides were purified with C18 cartridges. LC-MS/MS analysis was conducted on an EASY-nLC 1200 system coupled to an Orbitrap Exploris 480 mass spectrometer in data-independent acquisition (DIA) mode. Quality control parameters were as follows: peptide length was mainly distributed between 7–20 amino acids, consistent with trypsin digestion characteristics; the mass deviation of all identified peptides was <10 ppm, indicating high mass accuracy. DIA data were processed using DIA-NN (version 1.8) against the Mus musculus UniProt database (UP000000589), with a false discovery rate (FDR) threshold of 1% for both protein and peptide identification. Bioinformatic analyses included Gene Ontology (GO) annotation, Kyoto Encyclopedia of Genes and Genomes (KEGG) pathway enrichment, and protein–protein interaction network construction using the STRING database (confidence score > 0.7). Differentially expressed proteins with fold change > 1.5 and *p* < 0.05 were identified. Functional enrichment was assessed by Fisher’s exact test.

### 2.8. Statistical Analysis

Data are presented as mean ± standard error of the mean (SEM). The litter was treated as the independent experimental unit for all offspring assays; one female and one male pup were selected from each litter to avoid litter effect bias, and litter was included as a random effect in statistical models. For two-group comparisons, unpaired two-tailed Student’s *t*-test was used. For multi-group comparisons across three dietary groups, one-way ANOVA followed by Tukey’s post hoc test was applied to correct for multiple comparisons. Male and female offspring were analyzed separately to assess sex-specific effects. For repeated measurements (e.g., weekly body weight and blood glucose), repeated-measures ANOVA was used. For proteomic analysis, false discovery rate (FDR) < 1% was applied to correct for multiple testing in protein identification. Biochemical and behavioral endpoints were pre-specified confirmatory outcomes, and Tukey’s post hoc test was used for multiple comparison correction. All statistical analyses were performed using GraphPad Prism 9.0 software. Statistical significance was set at *p* < 0.05.

## 3. Results

### 3.1. Chemical Composition of LYT

Quantitative analysis revealed that LYT water extract contains substantial amounts of catechins (10.46 ± 0.48%), caffeine (3.71 ± 0.02%), and polysaccharides (6.27 ± 0.14%) (Table 1). Among catechins, EGCG (4.26 ± 0.27%) and GCG (2.11 ± 0.03%) were the most abundant. Theanine, the signature amino acid of tea, accounted for 0.69 ± 0.01% of the extract. These data provide the chemical basis for subsequent mechanistic investigations.

### 3.2. LYT Reduces Maternal BPA Body Burden, Potentially via Enhanced Glucuronidation and Excretion

**Figure 1:**
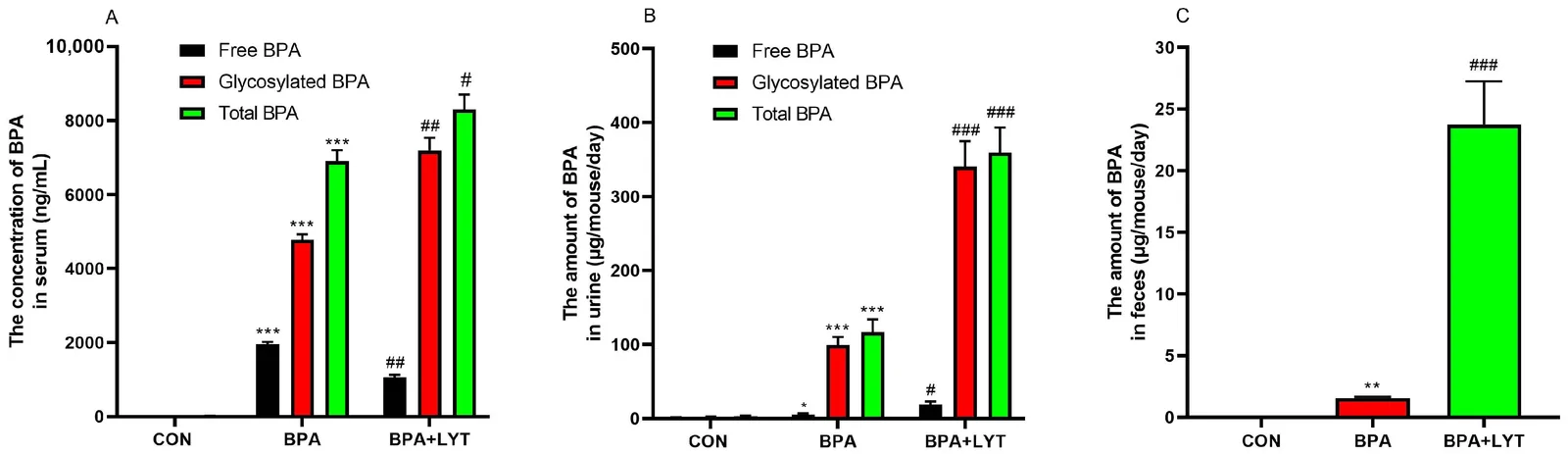
LYT reduces maternal BPA body burden and promotes excretion. (**A**) Serum free and conjugated BPA concentrations. (**B**) Urinary free and conjugated BPA concentrations. (**C**) Fecal free BPA content. Data are presented as mean ± SEM. *n* = 6 per group. Asterisks (*) indicate significant differences between Control and BPA groups (BPA effect); hash symbols (#) indicate significant differences between BPA and LYT + BPA groups (LYT intervention effect). * *p* < 0.05, ** *p* < 0.01, *** *p* < 0.001 vs. Control; # *p* < 0.05, ## *p* < 0.01, ### *p* < 0.001 vs. BPA group according to one-way ANOVA followed by Tukey’s post hoc test.

To determine whether LYT influences BPA toxicokinetics, we measured free and conjugated BPA in maternal serum, urine, and feces. Perinatal BPA exposure significantly elevated free BPA levels in all three compartments (Figure 1A–C). LYT supplementation markedly reduced serum free BPA while increasing conjugated (glucuronidated) BPA (Figure 1A), suggesting enhanced phase II glucuronidation metabolism. In urine, LYT increased both free and conjugated BPA excretion (Figure 1B), indicating facilitated elimination. Fecal free BPA was also elevated in the LYT group (Figure 1C), implying reduced intestinal absorption. Collectively, these results indicate that LYT reduces maternal BPA body burden through multiple potential pathways: limiting absorption, promoting glucuronidation metabolism, and enhancing urinary and fecal excretion. Direct measurement of UDP-glucuronosyltransferase (UGT) activity is needed in future studies to confirm the specific detoxification mechanism.

### 3.3. LYT Exerts Bidirectional Regulation of Glucose and Lipid Homeostasis Disrupted by Perinatal BPA Exposure

#### 3.3.1. Opposite Effects on Maternal and Offspring Glycemia

**Figure 2:**
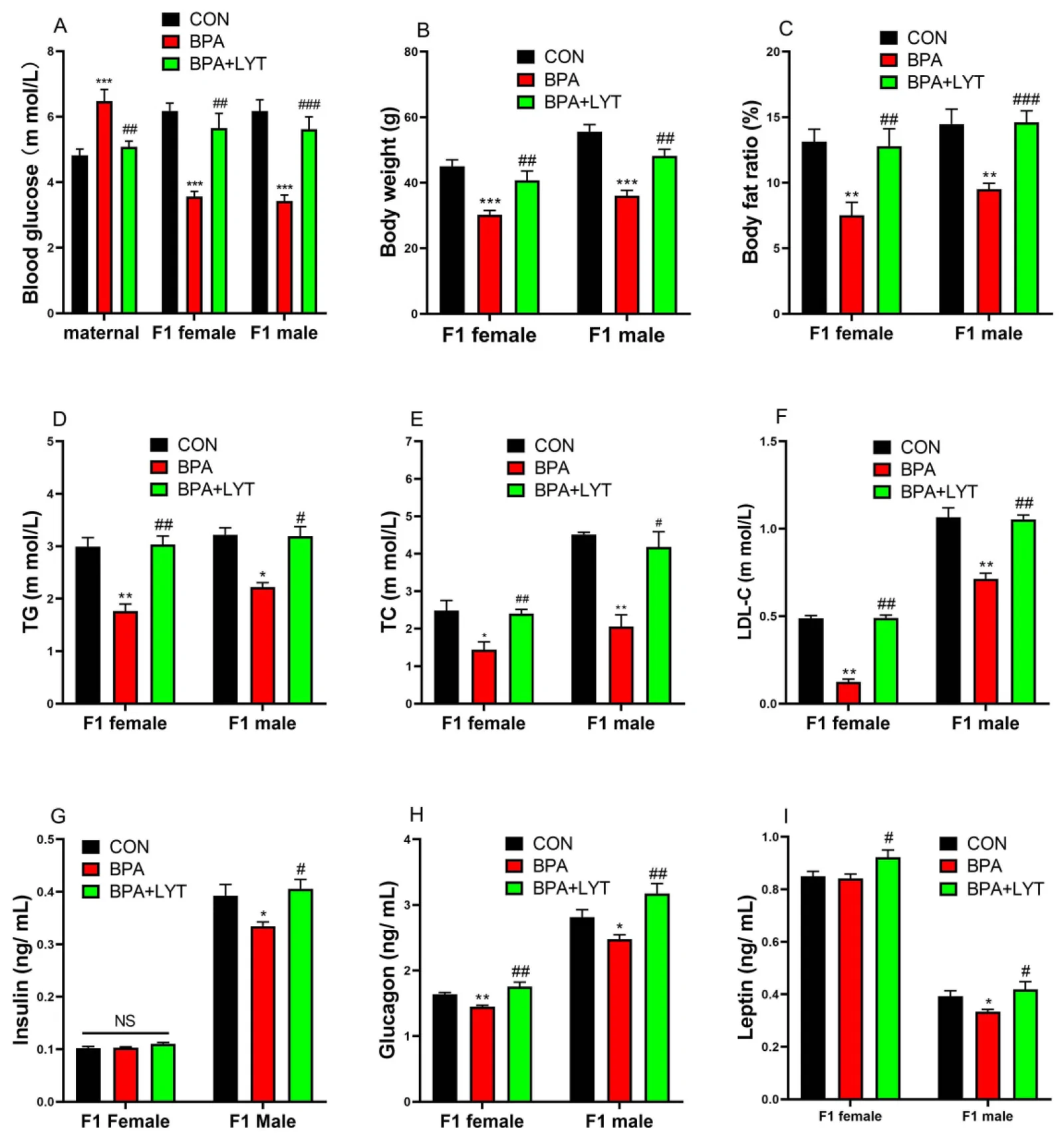
LYT bidirectionally regulates glucose and lipid homeostasis. (**A**) Fasting blood glucose in maternal mice (during gestation) and offspring (F1, 12 weeks). (**B**) Body weight of F1 mice at 24 weeks. (**C**) Body composition (fat mass and lean mass ratio) in F1 mice. (**D**–**F**) Serum TG, TC, and LDL-C levels in F1 mice. (**G**) Insulin in F1 mice. (**H**) Glucagon in F1 mice. (**I**) Leptin in F1 mice. Data are presented as mean ± SEM. *n* = 6 per group. Asterisks (*) indicate significant differences between Control and BPA groups (BPA effect); hash symbols (#) indicate significant differences between BPA and LYT + BPA groups (LYT intervention effect). * *p* < 0.05, ** *p* < 0.01, *** *p* < 0.001 vs. Control; # *p* < 0.05, ## *p* < 0.01, ### *p* < 0.001 vs. BPA group according to one-way ANOVA followed by Tukey’s post hoc test.

Perinatal BPA exposure exerted diametrically opposite effects on glucose homeostasis in mothers and offspring. In maternal mice, BPA induced significant hyperglycemia during gestation, with fasting blood glucose levels (6.48 ± 0.71 mmol/L) exceeding the diagnostic threshold for gestational diabetes (5.1 mmol/L) (Figure 2A). LYT supplementation completely normalized maternal blood glucose (5.08 ± 0.39 mmol/L).

Remarkably, the same BPA exposure that caused maternal hyperglycemia induced profound hypoglycemia in offspring (Figure 2A). F1 female and male mice from the BPA group exhibited significantly lower fasting glucose compared to controls. LYT intervention restored offspring glucose to normal levels, demonstrating that LYT can both lower hyperglycemia and raise hypoglycemia—a unique bidirectional regulatory capacity.

#### 3.3.2. LYT Normalizes Underweight and Hypolipidemia in Offspring

BPA-exposed offspring showed significant growth retardation, with reduced body weight in both sexes (Figure 2B). Body composition analysis revealed decreased fat mass and increased lean mass ratio (Figure 2C), indicating a shift toward leanness. Consistent with reduced adiposity, serum TG, TC, and LDL-C levels were markedly decreased in BPA-exposed offspring (Figure 2D,F). LYT supplementation fully restored body weight, body composition, and serum lipid profiles to control levels.

**Figure 3:**
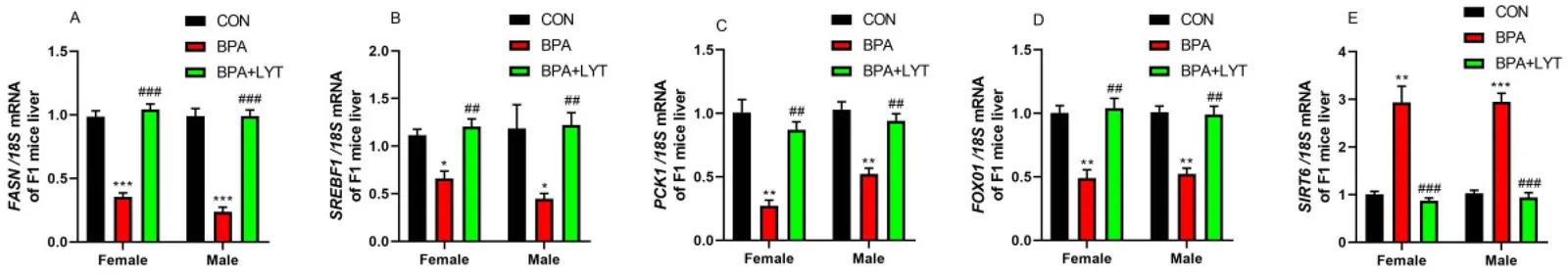
LYT modulates the mRNA expression of lipogenic and gluconeogenic genes in liver tissues of F1 mice. (**A**) *FASN*; (**B**) *SREBP1*; (**C**) *PCK1*; (**D**) *FOXO1*; (**E**) *SIRT6*. Data are presented as mean ± SEM. *n* = 6 per group. Asterisks (*) indicate significant differences between Control and BPA groups (BPA effect); hash symbols (#) indicate significant differences between BPA and LYT + BPA groups (LYT intervention effect). * *p* < 0.05, ** *p* < 0.01, *** *p* < 0.001 vs. Control; ## *p* < 0.01, ### *p* < 0.001 vs. BPA group according to one-way ANOVA followed by Tukey’s post hoc test.

To explore the molecular basis of this bidirectional regulation, we examined mRNA expression of key metabolic genes in the offspring’s liver. BPA exposure significantly downregulated gluconeogenic enzyme PCK1 and its transcriptional regulator FOXO1, while upregulating SIRT6, a negative regulator of FOXO1. In lipogenic pathways, BPA suppressed lipogenic enzyme FASN and its master regulator SREBP1, again with concurrent SIRT6 overexpression (Figure 3). LYT intervention reversed all BPA-induced gene expression abnormalities, restoring transcriptional levels of key gluconeogenic and lipogenic genes. These results suggest that the SIRT6/FOXO1 and SIRT6/SREBP1 pathways may be involved in the bidirectional metabolic regulation of LYT.

### 3.4. LYT Rectifies Hyperactivity and Reduces Energy Expenditure in Offspring Mice

#### 3.4.1. BPA-Induced Hyperactivity Is Normalized by LYT

**Figure 4:**
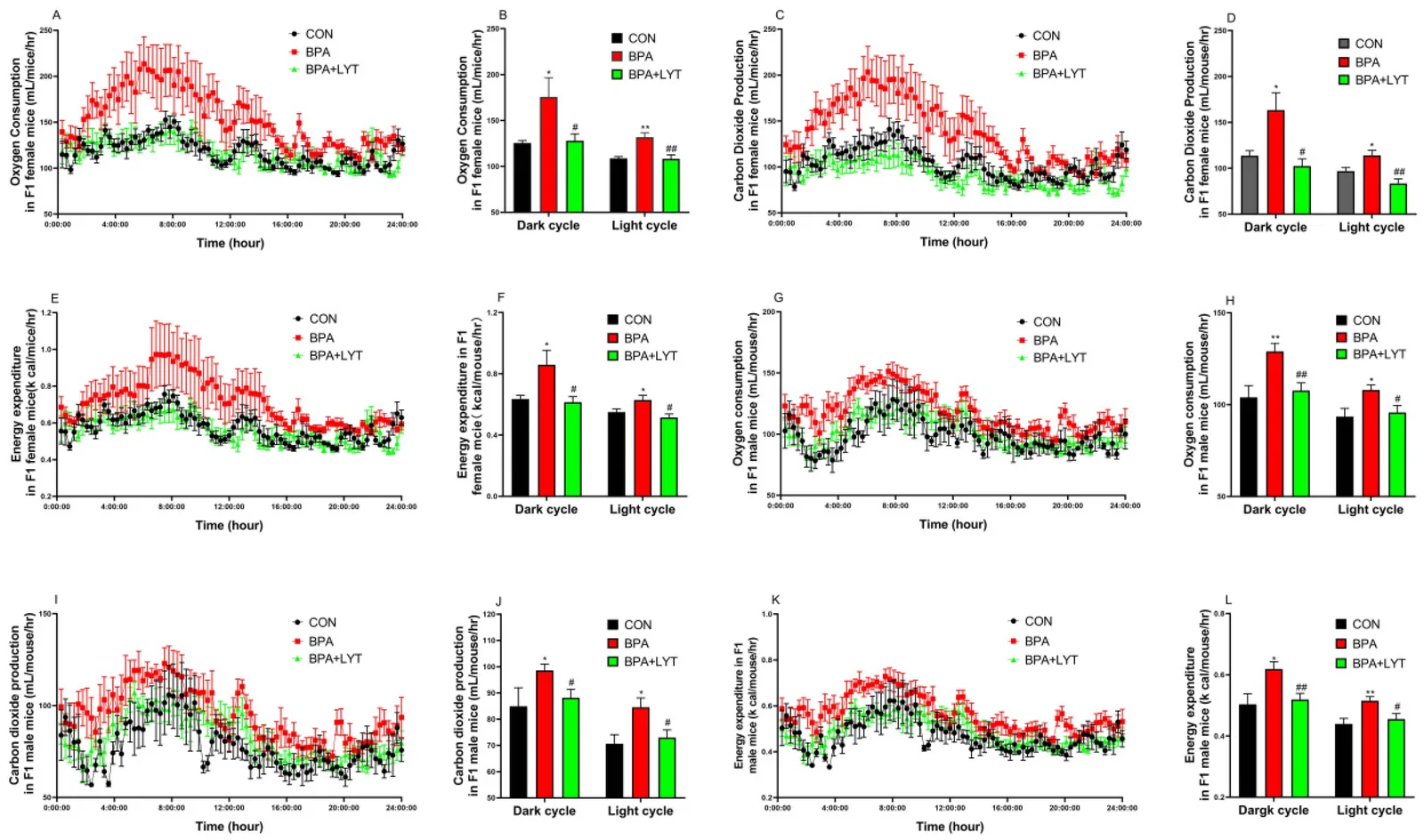
LYT normalizes energy expenditure. (**A**,**B**) Oxygen consumption (VO_2_), (**C**,**D**) carbon dioxide production (VCO_2_) and (**E**,**F**) energy expenditure in F1 female mice measured by CLAMS. (**G**,**H**) Oxygen consumption (VO_2_), (**I**,**J**) carbon dioxide production (VCO_2_) and (**K**,**L**) energy expenditure in F1 male mice measured by CLAMS. Data are presented as mean ± SEM. *n* = 6 per group.Data are presented as mean ± SEM. *n* = 6 per group. Asterisks (*) indicate significant differences between Control and BPA groups (BPA effect); hash symbols (#) indicate significant differences between BPA and LYT + BPA groups (LYT intervention effect). * *p* < 0.05, ** *p* < 0.01 vs. Control; # *p* < 0.05, ## *p* < 0.01 vs. BPA group according to one-way ANOVA followed by Tukey’s post hoc test.

Perinatal BPA exposure induced pronounced hyperactivity in offspring. Both female and male BPA-exposed mice showed significantly increased ambulatory, horizontal, and vertical activity during the light cycle (Figure 4). LYT supplementation completely normalized all activity parameters, restoring behavior to control levels.

#### 3.4.2. Hyperactivity Drives Increased Energy Expenditure

**Figure 5:**
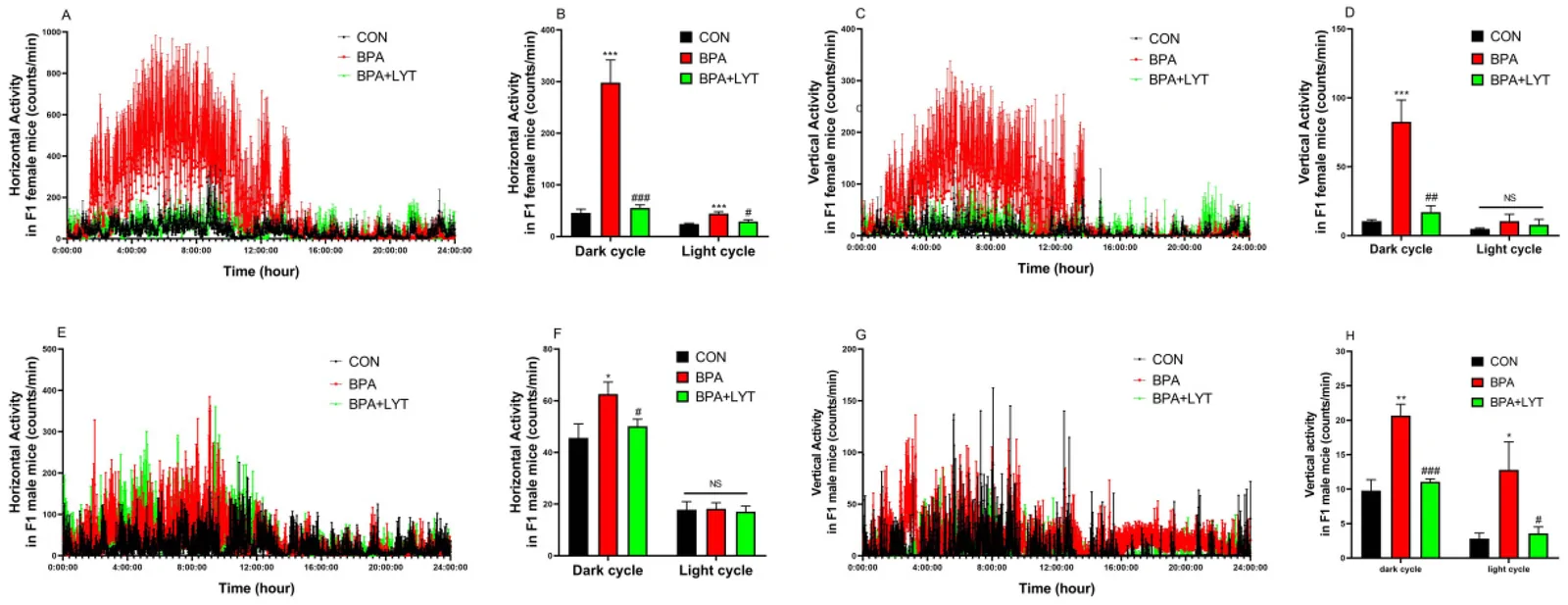
LYT rectifies BPA-induced hyperactivity. Spontaneous activity in F1 female (**A**–**D**) and male (**E**–**H**) mice measured by CLAMS. (**A**,**B**,**E**,**F**) Ambulatory and total horizontal activity. (**C**,**D**,**G**,**H**) Vertical activity. Data are presented as mean ± SEM. *n* = 6 per group. Asterisks (*) indicate significant differences between Control and BPA groups (BPA effect); hash symbols (#) indicate significant differences between BPA and LYT + BPA groups (LYT intervention effect). * *p* < 0.05, ** *p* < 0.01, *** *p* < 0.001 vs. Control; # *p* < 0.05, ## *p* < 0.01, ### *p* < 0.001 vs. BPA group according to one-way ANOVA followed by Tukey’s post hoc test.

Consistent with their hyperactive phenotype, BPA-exposed offspring exhibited elevated oxygen consumption (VO_2_) and energy expenditure during both light and dark cycles (Figure 5). Carbon dioxide production (VCO_2_) was similarly increased (Figure 5). 

LYT supplementation normalized all metabolic parameters, reducing VO_2_, VCO_2_, and energy expenditure to control levels. These data suggest that LYT’s metabolic benefits are partially mediated by behavioral normalization: alleviating hyperactivity reduces excessive energy demand, thereby facilitating the recovery of glucose and lipid homeostasis.

### 3.5. LYT Partially Restores BPA-Induced Dysregulation of Synaptic Pathways in Hippocampus

**Figure 6:**
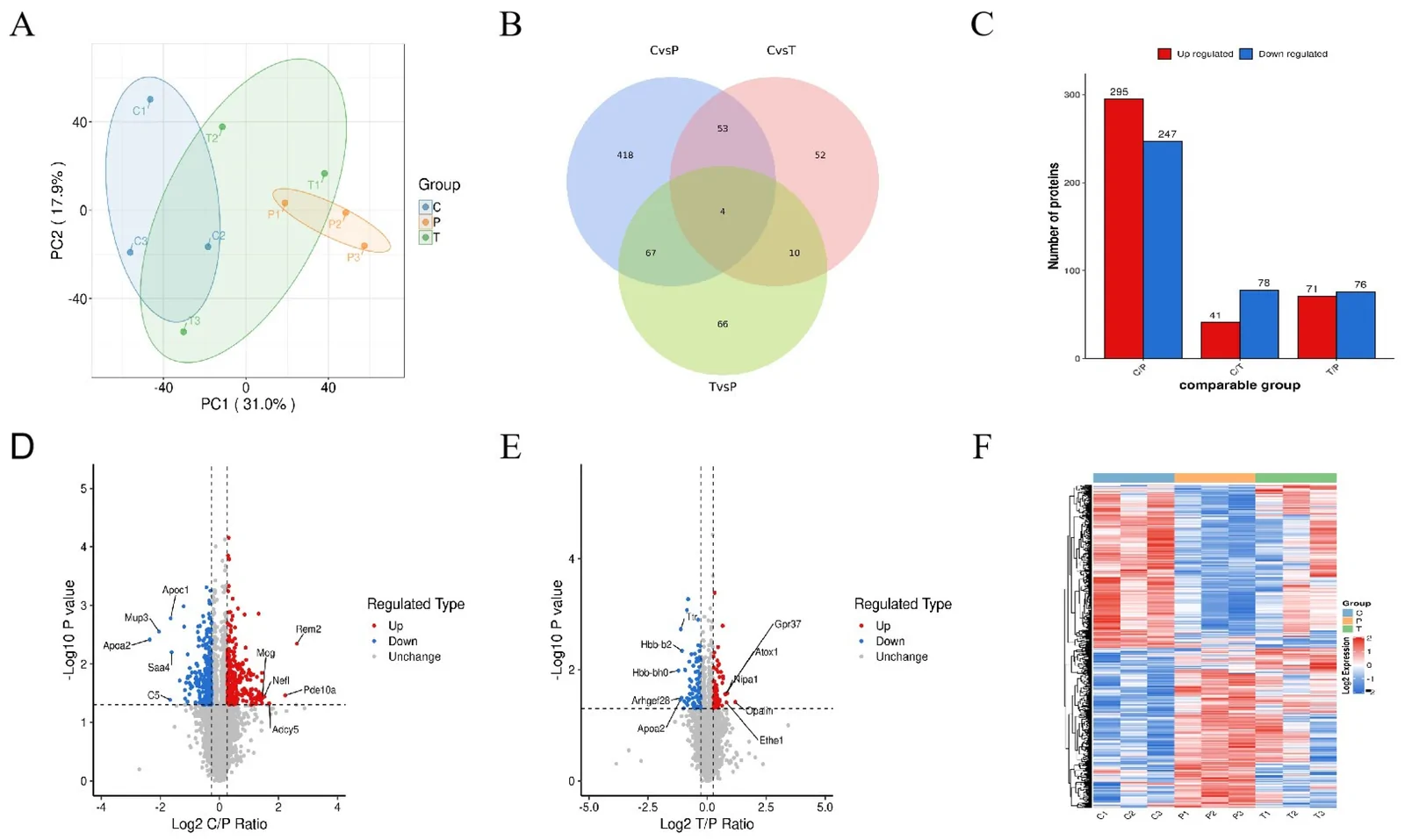
Sample correlation analysis and differential protein expression analysis in hippocampal tissue from female F1 mice. Hippocampal samples were collected from F1 female offspring, with *n* = 3 biological replicates per group (one mouse randomly selected from each of 3 independent litters out of 6 total F1 litters). The three groups are C (control group), P (BPA-induced group), and T (LYT intervention group). Two pairwise comparisons were performed: C vs. P (to identify BPA-induced changes, corresponding to asterisk notation in other figures) and T vs. P (to identify LYT intervention effects, corresponding to hash notation in other figures). (**A**) Principal component analysis (PCA) plot showing overall sample clustering; (**B**) Venn diagram of differentially expressed proteins showing overlap between the two comparisons; (**C**) statistical bar chart of upregulated and downregulated differential protein numbers in each comparison; (**D**) volcano plot of differential proteins in the C/P comparison (C vs. P); (**E**) volcano plot of differential proteins in the T/P comparison (T vs. P); (**F**) heatmap of differential protein expression across all samples. Data are presented as mean ± SEM. *n* = 3 biological replicates per group. Differentially expressed proteins with fold change > 1.5 and *p* < 0.05 according to unpaired Student’s *t*-test were identified.

Given the prominent hyperactive behavioral phenotype observed in female offspring, we performed label-free quantitative proteomic analysis on hippocampal tissues from female F1 mice to explore the underlying neural molecular mechanism (Figure 6).

**Figure 7:**
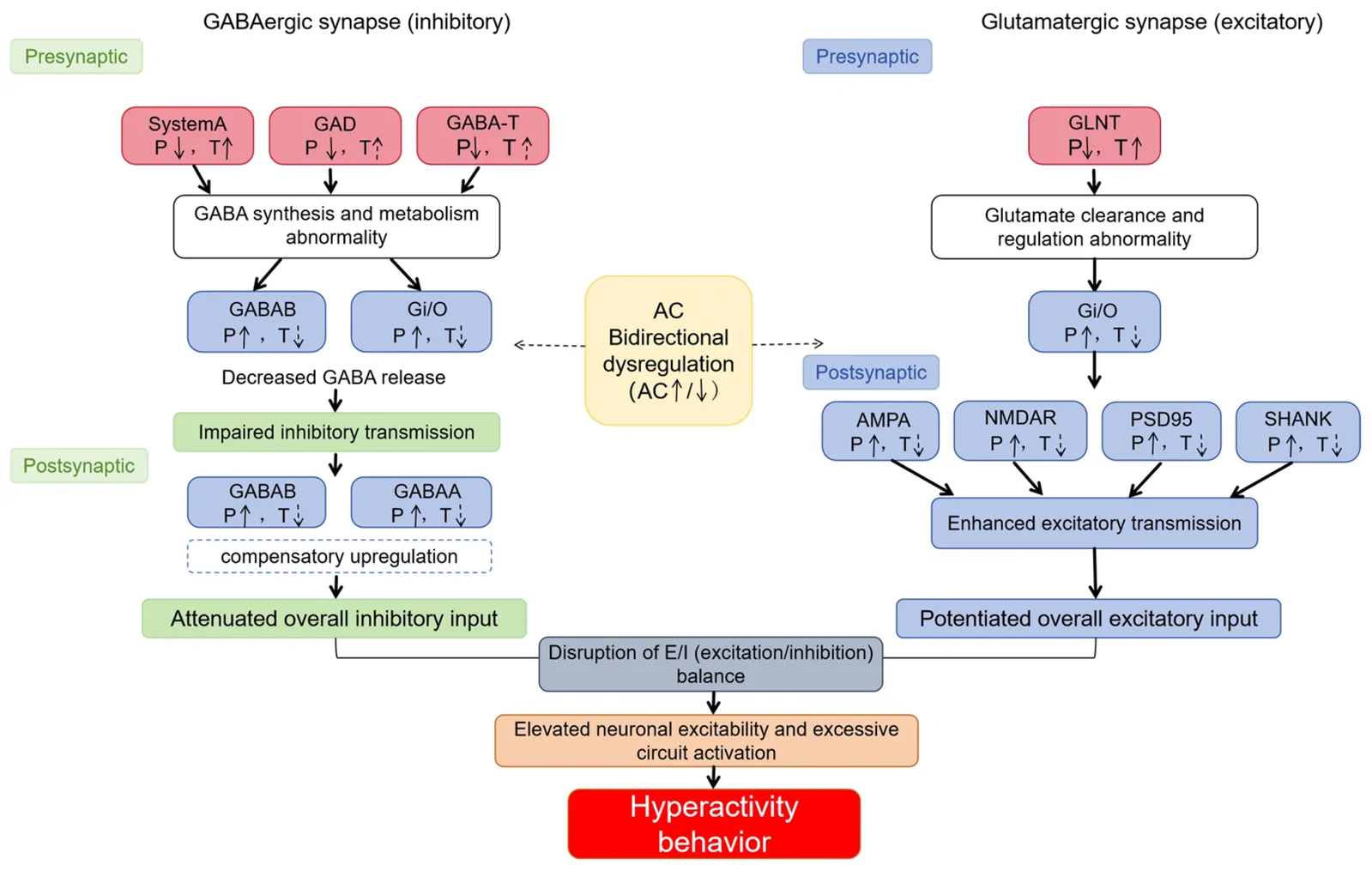
Potential mechanism underlying BPA-induced hyperactive behavior and the intervention effect of large yellow tea. This schematic working model is proposed based on the differential proteomics data from Figure 6, illustrating the putative molecular pathways through which BPA exposure induces hyperactivity and LYT intervention exerts protective effects. P↑/P↓ indicates upregulated/downregulated protein expression in the BPA group compared to the Control group (C vs. P comparison, corresponding to asterisk notation); T↑/T↓ indicates upregulated/downregulated protein expression in the LYT + BPA group compared to the BPA group (T vs. P comparison, corresponding to hash notation). Solid arrows indicate statistically significant differences (fold change > 1.5, *p* < 0.05), while dashed arrows indicate a recovery trend that did not reach statistical significance. All protein expression changes are derived from the same proteomics dataset as Figure 6 (*n* = 3 biological replicates per group, F1 female mouse hippocampus).

Functional annotation of differentially expressed proteins induced by BPA confirmed that the core dysregulated signaling pathways were glutamatergic synapse and GABAergic synapse pathways, which are systematically summarized in the self-organized pathway mechanism diagram (Figure 7).

BPA exposure triggered extensive dysregulation of glutamatergic and GABAergic synaptic signaling pathways. In the glutamatergic synapse pathway, the glutamine transporter GLNT was downregulated, limiting glutamate precursor supply, while postsynaptic AMPA/NMDA receptors and scaffolding proteins (PSD95, GKAP, SHANK) showed compensatory upregulation. Downstream signaling molecules were also dysregulated, with increased PLC and decreased PKC/PLD levels. In the GABAergic synapse pathway, glutamate decarboxylase (GAD), the key enzyme for GABA synthesis, was downregulated, reducing inhibitory neurotransmitter production. Concurrently, postsynaptic GABA-A and GABA-B receptors showed compensatory upregulation, while the GABA-degrading enzyme GABA-T was suppressed. LYT supplementation effectively corrected most abnormal protein expression patterns in both synaptic pathways.

The simultaneous disruption of excitatory glutamatergic and inhibitory GABAergic synaptic systems may destabilize neural excitation-inhibition (E/I) balance. The net effect of BPA-induced molecular dysregulation may favor excitatory neurotransmission, which is a potential pathological mechanism underlying the offspring hyperactivity. LYT-mediated partial restoration of synaptic protein expression may contribute to the normalization of BPA-induced abnormal behaviors, potentially via rebalancing E/I homeostasis. These findings are hypothesis-generating, and functional validation (e.g., electrophysiology, neurotransmitter quantification) in future studies is required to confirm E/I balance changes.

## 4. Discussion

This study provides the first evidence that maternal consumption of large yellow tea confers intergenerational protection against metabolic and behavioral disorders induced by perinatal BPA exposure. Our findings reveal that LYT acts through multiple potential interconnected mechanisms: (1) reducing BPA body burden by enhancing detoxification and excretion; (2) bidirectionally regulating glucose and lipid homeostasis via modulation of the SIRT6/FOXO1 and SIRT6/SREBP1 pathways; (3) normalizing hyperactivity and its associated excessive energy expenditure; and (4) partially restoring synaptic homeostasis in the hippocampus. Together, these mechanisms position LYT as a natural, multi-target antagonist of BPA toxicity with intergenerational protective effects.

### 4.1. LYT as a Natural Antagonist of BPA: Enhanced Detoxification as the Upstream Mechanism

The first line of defense against xenobiotic toxicity is efficient detoxification and elimination. Our results demonstrate that LYT reduces BPA body burden through multiple potential regulatory pathways: inhibiting intestinal BPA absorption (increased fecal free BPA), enhancing hepatic BPA glucuronidation (elevated serum conjugated BPA), and promoting urinary excretion of BPA metabolites (increased urinary total and conjugated BPA). This pattern suggests that LYT may induce UDP-glucuronosyltransferase (UGT) activity, enhancing BPA glucuronidation—the primary detoxification pathway in mammals [20,21,22].

Previous studies have confirmed that tea polyphenols represented by EGCG can modulate the activity of phase II detoxification enzymes [23]. The detoxification effect of LYT observed in this study may be attributed to a single active component or the synergistic effect of multiple ingredients including catechins, theanine, caffeine, and polysaccharides, which requires further verification. Notably, the LYT dosage adopted in this study (2.5% in diet, equivalent to approximately 10 g daily tea consumption in humans [24]) is half the effective dosage used in previous metabolic syndrome intervention models [4], suggesting that moderate LYT intake confers significant protective effects.

### 4.2. Bidirectional Metabolic Regulation via SIRT6-Centered Mechanisms

The most innovative finding of this study is the unique bidirectional homeostatic regulatory effect of LYT on BPA-disrupted glucose and lipid metabolism. LYT alleviated gestational hyperglycemia in maternal mice and rescued BPA-induced hypoglycemia, underweight and hypolipidemia in offspring. This homeostatic regulatory characteristic distinguishes LYT from single-target pharmacological drugs, which often exert unidirectional effects and carry potential adverse side effects.

Mechanistically, our transcriptional data suggest that SIRT6 acts as the core regulatory node of LYT’s bidirectional metabolic effects. As an NAD+-dependent deacetylase primarily localized to chromatin, SIRT6 exerts its metabolic regulatory functions predominantly at the transcriptional level, which is the well-recognized primary working mode of SIRT6 in modulating hepatic glucose and lipid metabolism [25,26,27]. It inhibits hepatic gluconeogenesis by promoting FOXO1 nuclear exclusion, thereby suppressing the transcriptional activation of gluconeogenic genes such as PCK1 [28]. In parallel, SIRT6 represses lipogenesis through histone deacetylation-mediated transcriptional inhibition of SREBP1 and its downstream lipogenic target genes including FASN [29,30]. Multiple studies have verified that SIRT6 governs the expression of SREBP1/2 and FOXO1 signaling cascades mainly via transcriptional repression and chromatin remodeling, rather than post-translational protein modification [25,26]. Consistently, the upstream regulatory role of SIRT6 in metabolic pathways is primarily manifested by altering the transcriptional abundance of downstream core genes, providing a solid theoretical basis for evaluating pathway activity through mRNA expression detection.

In the present study, perinatal BPA exposure triggered abnormal SIRT6 overexpression at the mRNA level, which persistently inhibited FOXO1 and SREBP1 transcriptional signaling, leading to suppressed gluconeogenesis and lipogenesis, and ultimately causing hypoglycemia and growth retardation in offspring. LYT intervention normalized SIRT6 transcriptional levels, relieved the inhibitory effect of SIRT6 on downstream pathways, and restored the dynamic balance of hepatic glucose and lipid metabolism. This SIRT6-dependent transcriptional regulatory mechanism explains the unique homeostatic characteristics of LYT, which repairs metabolic disorders rather than imposes fixed metabolic changes.

It should be acknowledged that the current mechanistic investigation has certain limitations: we only verified the transcriptional expression changes of core regulatory genes via RT-PCR, and did not further confirm the protein expression levels and post-translational modification of SIRT6, FOXO1, and SREBP1 by Western blotting. Nevertheless, existing authoritative studies have fully confirmed that the regulatory effects of SIRT6 on hepatic gluconeogenesis and lipogenesis pathways are mainly achieved through transcriptional-level gene regulation [25,26,27]. Therefore, the detected mRNA expression alterations of key pathway genes in this study can reliably reflect the activation status of the SIRT6-centered metabolic regulatory network. Future studies will supplement the current findings with protein-level validation and post-translational modification analysis to further refine and consolidate the mechanistic conclusions of this study.

Additionally, it is currently unclear whether LYT regulates SIRT6 expression directly through its active components, or indirectly by reducing BPA body burden. Both mechanisms are plausible: on one hand, LYT reduces BPA accumulation, thereby alleviating BPA-induced SIRT6 dysregulation; on the other hand, bioactive components of LYT such as catechins may directly modulate SIRT6 expression or activity. Further studies with separate experimental designs are needed to dissect these two potential regulatory pathways.

Another limitation is that this study did not include a LYT-only control group. Therefore, the effect of LYT on the normal physiological state requires further verification with a dedicated LYT-only group. This study focused on the protective effect of LYT under BPA exposure background, and the effect of LYT on the normal physiological state will be investigated in future studies.

### 4.3. Behavioral-Metabolic Interconnection: LYT Breaks the Hyperactivity-Energy Depletion Cycle

This study clarifies the critical correlation between BPA-induced behavioral abnormalities and metabolic disorders. Perinatal BPA exposure induces persistent hyperactivity in offspring mice, which significantly increases daily energy consumption. In the absence of compensatory increased food intake, excessive energy expenditure leads to reduced fat accumulation, insufficient energy supply, and subsequent hypoglycemia and lean phenotypes, forming a vicious cycle of hyperactivity-energy depletion-metabolic disorder. LYT fundamentally breaks this pathological cycle by normalizing offspring hyperactivity, reducing unnecessary energy consumption, and creating favorable conditions for the recovery of glucose and lipid homeostasis.

This behavioral-metabolic linkage provides potential insights into human health. Clinical studies have observed that children with attention-deficit/hyperactivity disorder (ADHD) often present abnormal growth and metabolic parameters [31,32], but the underlying mechanism remains unclear. Our findings in a mouse model provide a preliminary mechanistic basis for the comorbidity of hyperactivity and metabolic disorders, suggesting that targeted intervention for abnormal behaviors may improve metabolic health, and vice versa. However, these results are based on a mouse model with relatively high BPA exposure, and direct extrapolation to human populations should be done with caution.

### 4.4. Restoring Synaptic Homeostasis: A Potential Neural Mechanism Underlying Behavioral Improvement

Hippocampal proteomic analysis revealed the neural molecular mechanism of BPA-induced hyperactivity and LYT-mediated behavioral correction. BPA exposure causes consistent adaptive dysregulation in both excitatory glutamatergic and inhibitory GABAergic synaptic systems: reducing the synthesis of core neurotransmitters while inducing compensatory upregulation of postsynaptic receptors. This molecular mismatch may disrupt the dynamic balance of neural excitation and inhibition, and the net enhancement of glutamatergic excitatory transmission may drive hyperactive behaviors, consistent with the core pathophysiology of ADHD [33,34]. Clinical studies have also confirmed reduced GABA concentration and abnormal glutamate signaling in ADHD patients [35,36], further supporting the potential translational value of our findings.

Notably, LYT only partially restored the BPA-disrupted synaptic protein expression profile, but achieved complete normalization of behavioral phenotypes. This partial restoration phenomenon can be explained by multiple factors: first, LYT intervention only lasts throughout gestation and lactation, and the post-weaning protective effect is indirect, showing certain time and dose dependence; second, hippocampus is only one of the key brain regions regulating behavioral activity, and LYT may exert more comprehensive regulatory effects on other ADHD-related brain regions such as the prefrontal cortex; and third, partial correction of molecular pathway disorders is sufficient to reverse macroscopic functional and behavioral abnormalities. This also reflects the multi-target collaborative regulatory advantage of natural dietary interventions.

Notably, these conclusions are based on proteomic expression changes, and we have not performed electrophysiological recordings or neurotransmitter concentration measurements to directly verify functional E/I balance alterations. Therefore, the neural mechanism proposed here remains hypothesis-generating and requires functional validation in future studies. The exact mechanism through which BPA and LYT regulate hippocampal protein expression also remains to be elucidated. On one hand, BPA may exert direct neurotoxic effects by crossing the blood–brain barrier, leading to dysregulation of synaptic protein expression; LYT may reduce BPA body burden and thus indirectly alleviate neurotoxicity. On the other hand, bioactive components of LYT (such as catechins and theanine) may cross the blood–brain barrier and directly regulate neural signaling pathways. Further studies are needed to distinguish between these direct and indirect mechanisms.

### 4.5. Potential Mechanisms of Intergenerational Effects and Future Directions

The persistent protective effect of maternal LYT supplementation on offspring after weaning indicates genuine intergenerational protection rather than direct drug intervention in offspring. The intergenerational protective mechanism of LYT may involve three aspects: first, LYT reduces maternal BPA accumulation and transmission during gestation and lactation, reducing toxic stimulation of fetal and neonatal developing organs during critical developmental windows; second, BPA exposure induces heritable epigenetic modifications including DNA methylation and histone modification [37,38], and LYT may alleviate BPA-induced epigenetic aberrations to maintain long-term metabolic and neural homeostasis; and third, maternal metabolic health regulated by LYT improves placental function and milk composition, providing a healthy developmental environment for offspring [39].

Future research will focus on five core directions to further improve the research system: (1) investigate the persistence of LYT’s protective effects in F2/F3 generations and the underlying epigenetic mechanisms; (2) clarify the dose–response relationship of LYT intervention and identify its core active protective components; (3) analyze the molecular mechanism of sex-specific differences in BPA toxicity and LYT protection; (4) expand brain region research to explore the regulatory effect of LYT on prefrontal cortical neural function; and (5) verify whether LYT exerts protective effects against common BPA substitutes (BPS, BPF).

## 5. Conclusions

This study demonstrates that maternal LYT consumption confers intergenerational protection against BPA-induced metabolic and behavioral disorders through multiple potential mechanisms: enhanced toxin clearance, bidirectional SIRT6-mediated glucose and lipid metabolic regulation, hyperactivity correction, and partial restoration of hippocampal synaptic homeostasis. These findings establish LYT as a promising dietary strategy for mitigating environmental health risks during development, providing new insights and a theoretical basis for the development of natural functional foods to protect against environmental toxicant damage.

## Figures and Tables

**Table 1:** The contents of catechins, caffeine, total amino acids and polysaccharides in water-soluble component of LYT measured by UPLC analysis.

Compound	LYT (%)
EGCG	4.26 ± 0.27
GCG	2.11 ± 0.03
EC	1.68 ± 0.19
GC	1.38 ± 0.02
C	0.92 ± 0.01
Total catechins	10.46 ± 0.48
Theanine	0.69 ± 0.01
Total amino acids	1.68 ± 0.01
Caffeine	3.71 ± 0.02
Polysaccharide	6.27 ± 0.14

Abbreviations: (−)-epigallocatechin gallate (EGCG), (−)-gallocatechin gallate (GCG), (−)-epicatechin (EC), (+)-gallocatechin (GC), (+)-catechin (C). Total catechins is the sum amount of EGCG, EC, GCG, GC, and C; total amino acids are the amount of theanine plus other free amino acids. LYT, large yellow tea.

## Data Availability

The Supplementary Materials (primer sequences for qPCR) are openly available in Zenodo. The remaining raw experimental data are available on request from the corresponding authors.

## Supplementary Materials

The following supporting information can be downloaded at: https://www.mdpi.com/article/10.3390/metabo16080588/s1, Figure S1. Biochemical index of maternal mice; Figure S2. Biochemical index of offspring mice; Figure S3. The mRNA expression of genes involved in lipolysis and gluconeogenesis in the liver of F1 mice. Table S1. Primer sequences used for RT-PCR gene expression experiment.
