# Supplementary material for: Maternal Large Yellow Tea Supplementation Confers Intergenerational Protection Against BPA-Induced Metabolic and Behavioral Disorders in Mice
Source: Metabolites. 2026 Aug 18;16(8):588. doi: 10.3390/metabo16080588 (PMC13515447; doi:10.3390/metabo16080588)
Supplement: Supplementary file 1 [file metabolites-16-00588-s001.zip › Table S1.pdf]

Table S1. Primer sequences used for RT-PCR gene expression experiment

	Primer Sequences	
Genes	Forward (5′–3′)	Reverse (5′–3′)
18S	AGA AACGGCCACATCCAA	GGGTCGGGAGTGGGTAAT TT
Fasn	CGTGTGACCGCCATCTATATC G	TGAGGTTGCTGTCGTCTGTAGTCTT
Srebf1	AGTCCAGCCTTTGAGGATAGC C	CCGTAGCATCAGAGGGAGTGA G
Adipor2	CCTTTCGGGCCTGTTTTAAGA	GAGTGGCAGTACACCGTGTG
Cpt1a	CTTCAAAAACAGCAAGATAGGCATA	TTACAGTGTCCATCCTCTGAGTAGC
Pparg	GAAAGACAACGGACAAATCACCAT	CGGCTTCTACGGATCGAAACTG
PCK1	GAGAAAGCGTTCAATGCCAG	ATGCCGATCTTTGACAGAGG
Sirt6	GGCTACGTGGATGAGGTGAT	GGCTTGGGCTTATAGGAACC
Fox01	GAGTGGATGGTGAAGAGCGT	TGCTGTGAAGGGACAGATTG
G6pc	CGACTCGCTATCTCCAAGTGA	GGGCGTTGTCCAAACAGAAT
