# Supplementary material for: Maternal Large Yellow Tea Supplementation Confers Intergenerational Protection Against BPA-Induced Metabolic and Behavioral Disorders in Mice
Source: Metabolites. 2026 Aug 18;16(8):588. doi: 10.3390/metabo16080588 (PMC13515447; doi:10.3390/metabo16080588)
Supplement: Supplementary file 1 [file metabolites-16-00588-s001.zip › Supplementary Figure legends.pdf]

Figure S1. Biochemical index of maternal mice. A. Food intake in maternal mice. B. Water intake in maternal mice. C. Body weight of maternal mice. There are no differences significantly in food and water intake and body weight between different groups in maternal mice. Data are the means ±SEM. n=6 for each group.

Figure S2. Biochemical index of offspring mice. A. Food intake in maternal mice. B. Water intake in maternal mice. C. GLP-1 in serum. D. Glycogen in liver. data are the means ± SEM, n = 6 for each group. There are no differences in food and water intake, glucagon and glycogen in different groups significantly. Data are the means ±SEM. n=6 for each group.

Figure S3. The mRNA expression of genes involved in lipolysis and gluconeogenesis in the liver F1 mice. A. Cpt1α B. Adipor2; C. G6pc. Thera were no differences in the mRNA expression of Cpt1α significantly between the control and BPA group, though BPA exposure increased the mRNA expression of Cpt1α compared with the control group in F1 mice. Data are the means ±SEM. n=6 for each group.
